# Circadian rhythms in parasites

**DOI:** 10.1371/journal.ppat.1006590

**Published:** 2017-10-12

**Authors:** Filipa Rijo-Ferreira, Joseph S. Takahashi, Luisa M. Figueiredo

**Affiliations:** 1 Department of Neuroscience, University of Texas Southwestern Medical Center, Dallas, Texas, United States of America; 2 Howard Hughes Medical Institute, The University of Texas Southwestern Medical Center, Dallas, Texas, United States of America; 3 Instituto de Medicina Molecular, Faculdade de Medicina, Universidade de Lisboa, Lisboa, Portugal; Buffalo, UNITED STATES

Circadian rhythms are 24-hour physiological oscillations found at all levels of organization from gene expression to behavior. They have been described in organisms across the tree of life, from bacteria to humans. Photosynthesis in plants and sleep/wake cycles in animals, are 2 examples of circadian rhythms.

## What parameters characterize a circadian rhythm?

**Fig 1:**
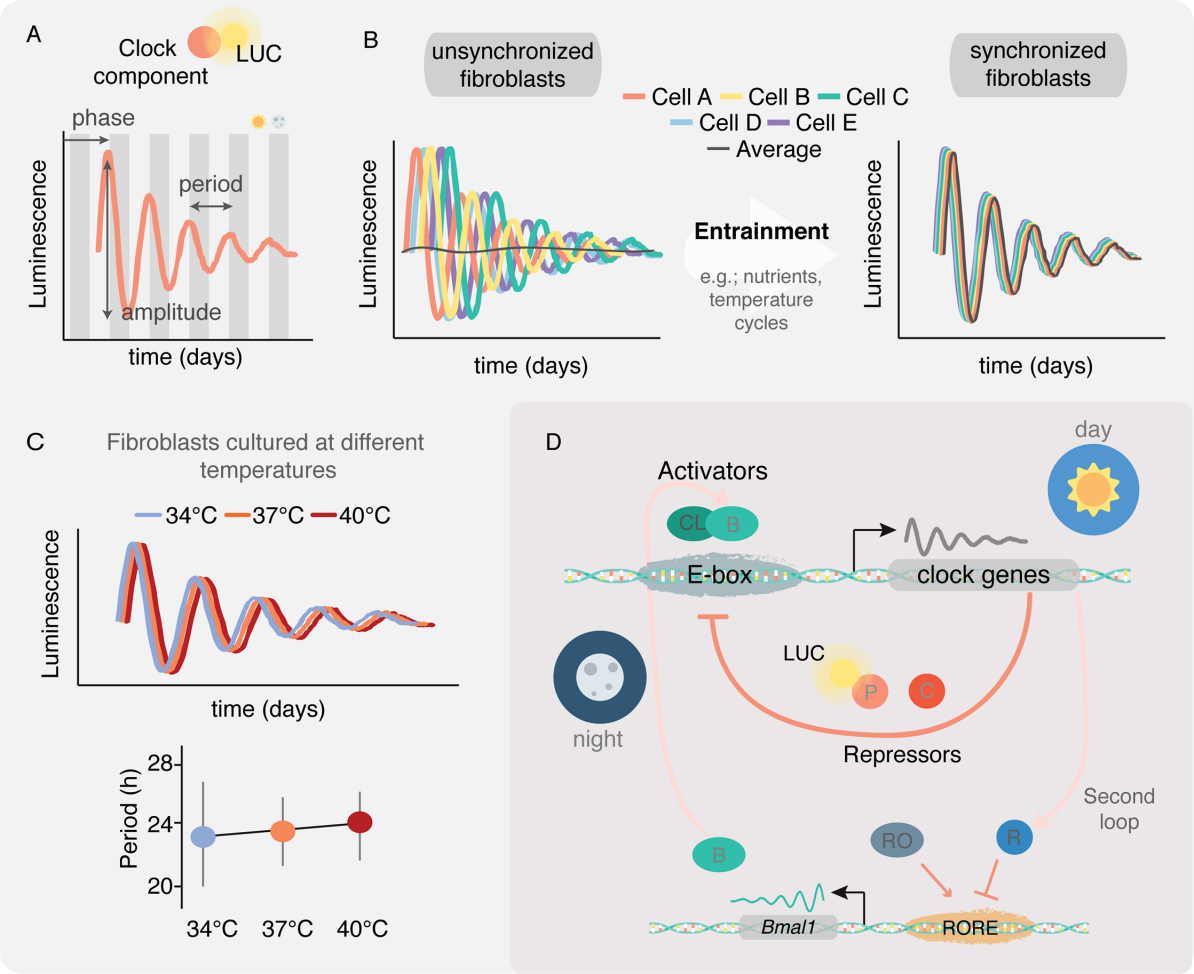
Properties of circadian clocks. **A.** Bioluminescence rhythms of a tissue explant, measured by light emission of Luciferase (LUC) fused with the clock component PERIOD2. Period, phase, and amplitude can be estimated from such rhythms. Gray shading represents the projected subjective day/night cycles (based on the pattern that the animal was exposed to prior to the start of the explant culture) given that cultures are maintained in constant conditions. **B.** When cultured without any external stimuli for many days, individual fibroblasts retain oscillations but they become asynchronous. To obtain a synchronized population of fibroblasts, entrainment cues need to be given. Once synchronized, the population of fibroblasts will cycle coherently even in the absence of any stimuli for approximately 1 week (depending on the entrainment signals). **C.** Bioluminescence at 3 different temperatures. The period of the circadian clock is maintained, highlighting that circadian rhythms are temperature compensated. Plot adapted from the experiments of Bieler & Cannavo [7]. **D.** Core molecular components of the mammalian circadian clock. CL represents CLOCK, B represents BMAL1, C represents CRYPTOCHROMEs, and P represents PERIODs proteins. A common circadian reporter consists of Luciferase (LUC) fused to PERIOD2 protein, one of the clock repressors. In the second loop involving the nuclear receptors, R represents REV-ERBs (α, β) and RO represents RAR-related orphan receptors RORs (α, β, γ). RORE represents receptor-related orphan receptor response elements.

Rhythms are self-sustained 24-hour oscillations. In such oscillations, we can measure phase, amplitude, and period (Fig 1A). Mathematical algorithms help us estimate such values from high-throughput data [1, 2].

## What defines a circadian rhythm?

Not all daily rhythms are circadian rhythms. The earth’s rotation exposes organisms to rhythmic changes in the environment, such as light/dark cycles. These environmental changes impose rhythms in organisms; however, many of these rhythms may not persist when in constant conditions. The 24-hour rhythms that do persist in these constant conditions are circadian rhythms. For a process to be defined as a circadian rhythm, it must fulfill 3 properties: i) have a periodicity of around 24 hours (± 2 hours), even in the absence of environmental cues (free-running period); ii) be able to readjust its timing (phase and period) in response to environmental cues (entrainment); iii) and maintain an approximately 24 hour period at different temperatures within the organism’s physiological range (temperature compensation of the period) (Fig 1A–1C).

## What is entrainment? What are free-running conditions? and what is temperature compensation?

To answer these questions, we will refer to studies with fibroblasts as an example. Entrainment is the process by which circadian rhythms become synchronized to a cyclic environmental cue. Because each individual cell has a slightly different period, rhythms of fibroblast cultures become asynchronous [3] when cultured in constant (free-running) conditions for many days. However, when environmental cues, such as nutrients (serum, medium change), hormones (glucocorticoids), or temperature cycles are given periodically, the circadian rhythms of the population synchronize (entrain) [4–6] (Fig 1B). These rhythms maintain their period when at constant 34°C, 37°C, and 40°C, which shows the temperature compensation of circadian rhythms [6, 7] (Fig 1C).

## What drives the circadian rhythms?

In mammals, circadian rhythms are regulated by intricate loops that at their core involve 2 transcription factors: CLOCK and BMAL1. These transcription factors activate the transcription of their own repressors, PERIOD (PER) and CRYPTOCHROME (CRY) proteins, among many other downstream genes involved in several physiological pathways (reviewed in [8]) (Fig 1D). A second loop involves the nuclear receptors REV-ERBs (α, β, also known as nuclear receptor subfamily 1, group D, members 1 and 2) and RAR-related orphan receptors RORs (α, β, γ, Retinoic Acid-Related Orphan Receptors). REV-ERBs transcription is driven by CLOCK:BMAL1. These nuclear receptors translocate back into the nucleus and bind to receptor-related orphan receptor response elements (ROREs) in the promoter of *Bmal1*, repressing its expression. RORs, on the contrary, activate *Bmal1* transcription through binding to the same elements on its promoter (Fig 1D). These same nuclear receptors modulate the expression of the nuclear factor interleukin (*Nfil3*), which in turn regulates RORs expression, forming a third loop. The second and third loops play important roles in immune response [9–11], regulating, for example, the timing of proinflammatory T_h_17 cell differentiation [12].

## How can we detect circadian rhythms at the molecular level?

Fibroblast cultures have been widely studied to investigate the properties of circadian rhythms. These cells, as do virtually all cells in the animal body, have cell-autonomous circadian rhythms that can be monitored using luciferase reporters [13, 14] (Fig 1A and 1D) or fluorescent proteins [4] driven by cycling clock genes.

At the population level, it is also possible to detect circadian rhythms using genome-wide technologies: RNA-sequencing identified approximately 20% of genes oscillating in the mouse liver; ChIP-seq revealed rhythms in the epigenetic and transcriptional patterns [15]; Metabolomic analysis revealed oscillations of many metabolites [16]; and Proteomics and Phosphoproteomics also identified circadian oscillations in protein abundance and their phosphorylation status [17–19].

## Have daily rhythms been detected in parasites?

Many parasitic infections show rhythmic daily patterns. Malaria blood-stage parasites have a synchronous asexual cycle, with a coordinated cycle from the moment of invasion of the red blood cells until their bursting. This cycle lasts 24 hours or multiples of 24 hours, depending on the species [20], and is associated with recurrent fevers in the host. The human infectious stage of the *Schistosoma mansoni* parasite (known as cercariae forms) emerges from snails and swims in fresh water to infect humans by penetrating through the skin. Interestingly, the emergence of this infectious stage is rhythmic and matches the behavior of its final host: occurring during the daytime in parasites that infect humans and in the early evening in parasites that infect nocturnal rats [21, 22]. For filarial parasites, the appearance of the transmissible form in the blood is also rhythmic, with their higher numbers matching the vector feeding pattern [23]. A rhythmic number of parasites in the blood is common to many other parasite species. The number of *Trypanosoma rotatorium* in the blood of the frog [24] and *Trypanosoma congolense* and *Trypanosoma lewisi* in the blood of rodents [25, 26] varies throughout the day.

Despite these and many other examples of rhythmic patterns in parasitic infections, until recently we did not know if these behaviors were intrinsic to the parasite or whether parasites were simply responding to rhythmic environmental cues of their host.

## What did we learn about *Trypanosoma brucei* circadian rhythms?

*T*. *brucei* is the parasite responsible for causing sleeping sickness in humans. Although no daily oscillation in parasite number has been detected in *T*. *brucei*-infected animals [25], we have recently shown that in culture, these extracellular parasites have intrinsic circadian rhythms in gene expression [27]. Such rhythms were identified by initially entraining the parasite population with temperature cycles followed by assessing the transcriptome by RNA-seq of samples every 4 hours for 2 days. Approximately 10% of the transcriptome cycles in free-running conditions in 2 life cycle stages of *T*. *brucei* (bloodstream and insect forms). Many of the cycling genes encode proteins involved in metabolic pathways and the overall metabolic activity of the population is indeed synchronous, with intracellular ATP oscillating during the day. This finding suggests that other parasites may also possess endogenous circadian rhythms.

## Are all circadian rhythms dependent on a transcription-translation feedback loop?

Most circadian timekeeping mechanisms rely on a transcriptional-translation feedback loop [28]. As mentioned above, in mammals, CLOCK and BMAL1 heterodimers rhythmically bind to promoters across the genome, activating their transcription [15]. This transcriptional-translation feedback loop mechanism of the clock was first described in pioneering studies in fruit flies [29] and fungi [30]. Curiously, in cyanobacteria, the circadian clock can be reconstituted in vitro: the three KaiABC proteins with ATP are sufficient to generate a temperature-compensated circadian KaiC phosphorylation cycle [31], which could potentially suggest that transcription is not important to drive this clock. However, even though transcription and translation of *kai* genes are not essential for the generation of this KaiC phosphorylation rhythm, feedback regulation of *kaiBC* transcription appears to be vital for maintaining robust circadian rhythms in vivo [32, 33].

Despite the importance of the transcription-translation feedback loop, there are many other layers of regulation driving circadian rhythms. Posttranscriptional [34] and posttranslational regulation [35], as well as the redox state of NAD (nicotinamide adenine dinucleotide) cofactors [36, 37] have been shown to be important to tune the rhythms. In *T*. *brucei*, transcription is believed to be constitutive and gene expression regulated mostly at the posttranscriptional level [38, 39]. The circadian clock in this parasite appears to also be driven mainly by posttranscriptional mechanisms because polycistronic units (PCUs) are not synchronous and individual transcripts within a PCU can be noncyclic or cyclic with different phases of transcript levels [27].

## Is entrainment by light necessary to establish circadian rhythmicity?

Light is undoubtedly the strongest and most well studied environmental cue on our planet. It can be perceived directly by photoactive proteins (phytochrome and cryptochrome in plants) [40] and by specialized photoreceptors in the retina (mammals) [41], or indirectly via the redox status of the cell (cyanobacteria) [42]. In fact, highlighting how important light is for life on Earth, photoreceptors have independently evolved multiple times throughout evolution [43]. However, the rhythmicity of the Earth imposes not only light/dark cycles but also temperature and humidity cycles. Plant circadian rhythms can entrain to temperature cycles, in which day and night temperatures differ by 4°C or less [44]. So far ex vivo, peripheral mammalian clocks appear to be irresponsive to light. However, when in culture, fibroblasts, red blood cells, lung, liver, and kidney are very sensitive to temperature changes [5, 45–48]. They can entrain to low-amplitude temperature cycles that mimic the range of circadian variation of body temperature rhythms.

Parasites, by living inside a host, may have evolved similar entrainment mechanisms to a mammalian peripheral clock, since throughout their life cycle parasites also have little exposure to light. We showed that *T*. *brucei* parasites (both mammalian-bloodstream and insect-procyclic forms) are able to entrain to temperature cycles differing by only 5°C, whereas the bloodstream forms do not appear to entrain to light/dark cycles [27]. It is possible, however, that—similar to peripheral clocks—other stimuli can also entrain these and other parasites, such as nutrient availability [49], hormones [50, 51], and perhaps even rhythmic immune pressure [52].

## What are the selective advantages for parasites to have a circadian clock?

Having circadian clocks is advantageous for organisms. Their longevity and growth rate are improved when these organisms are in a daily environment with a period that resonates with their endogenous circadian clock. Plants, by being able to anticipate the daily changes of their environment, have increased growth, survival, and competitive advantage [53]. In cyanobacteria, the clock mutants whose circadian clock has a shorter period than 24 hours, are less fit to live in a 24-hour day [54]. When mice are exposed to chronic jetlag, and therefore their circadian clock continuously disrupted, they have a shorter lifespan [55].

*T*. *brucei* also has a circadian clock—it’s able to generate circadian rhythmic gene expression in the absence of host cells and in the absence of any stimuli [27]. What is this rhythm for? Why does *T*. *brucei* invest its energy in making sure some mRNAs are more abundant at certain times of the day [56]? We observed that many of the genes that undergo circadian expression encode for metabolic enzymes. Inside the mammalian host, nutrient availability also cycles throughout the day. Thus, it is possible that the circadian clock of the parasite coordinates its metabolism to the predictable circadian nutrient changes.

Additionally, anticipating the rhythmic immune response could be beneficial for the parasites. The number of leukocytes in the mouse circulation varies across the day, being mostly recruited into the tissues at the beginning of the mouse active phase [57]. Also at this time there is higher cytokine release when mice are challenged with lipopolysaccharide (LPS) [9]. These, and other, examples show that immune responses are different throughout the day (reviewed in [58]).

Among many possible advantages, anticipating the optimal timing for transmission would be quite an important advantage for a parasite. The vector biting habits for a blood meal follow a circadian rhythm [59–61], and it would be beneficial to have the transmissible forms ready in circulation to match this vector behavior and therefore more efficiently complete the life cycle. In fact, a mismatch in timing between parasite and host rhythms is costly to the malaria parasite replication and transmission [62]. As described above, in some parasitic infections the appearance of transmissible forms follows a rhythmic pattern [21–23], what is still unknown is whether this is driven by an intrinsic circadian clock of the parasite.

## Where do we go from here?

It would be interesting to assess if other parasites also have endogenous circadian clocks. In *T*. *brucei*, the next step is to identify the molecular timekeeping machinery responsible for driving the circadian oscillations. By disrupting the expression of such molecules and thus perturbing this mechanism, one could test the physiological consequences and possible evolutionary advantages for the parasite in keeping a circadian clock.
